# Psychosocial, behavioral and clinical correlates of children with overweight and obesity

**DOI:** 10.1186/s12887-020-02145-2

**Published:** 2020-06-10

**Authors:** Vidhu V. Thaker, Stavroula K. Osganian, Sarah D. deFerranti, Kendrin R. Sonneville, Jennifer K. Cheng, Henry A. Feldman, Tracy K. Richmond

**Affiliations:** 1 Division of Molecular Genetics, Department of Pediatrics, Columbia University Irving Medical Center, 1150, St. Nicholas Avenue, New York, NY 10032 USA; 2 National Institute of Diabetes and Digestive and Kidney Diseases, National Institutes of Health, Bethesda, MD 20892 USA; 3 Division of Ambulatory Cardiology, Deparment of Cardiology, Boston Children’s Hospital, Boston, MA 02115 USA; 4 Department of Pediatrics, Harvard Medical School, Boston, MA 02115 USA; 5 University of Michigan School of Public Health, Ann Arbor, MI 48109 USA; 6 Division of Primary Care, Boston Children’s Hospital, Boston, MA 02115 USA; 7 Institutional Centers for Clinical and Translational Research, Boston Children’s Hospital, Boston, MA 02115 USA; 8 Division of Adolescent/Young Adult Medicine, Boston Children’s Hospital, Boston, MA 02115 USA

**Keywords:** Childhood obesity, Psychosocial, Behavioral

## Abstract

**Background:**

Psychological and behavioral correlates are considered important in the development and persistence of obesity in both adults and youth. This study aimed to identify such features in youth with severe obesity (BMI ≥ 120% of 95^th^percentile of sex-specific BMI-for-age) compared to those with overweight or non-severe obesity.

**Methods:**

Youth with BMI ≥ 85^th^ percentile were invited to participate in a prospective research registry where data was collected on attributes such as family characteristics, eating behaviors, dietary intake, physical activity, perception of health and mental well-being, and cardiometabolic parameters.

**Results:**

In a racially/ethnically diverse cohort of 105 youth (65% female, median age 16.1 years, range 4.62–25.5), 51% had severe obesity. The body fat percent increased with the higher levels of obesity. There were no differences in the self-reported frequency of intake of sugar sweetened beverages or fresh produce across the weight categories. However, the participants with severe obesity reported higher levels of emotional eating and eating when bored (*p* = 0.022), levels of stress (*p* = 0.013), engaged in fewer sports or organized activities (*p* = 0.044), and had suboptimal perception of health (*p* = 0.053). Asthma, depression and obstructive sleep apnea were more frequently reported in youth with severe obesity. The presence of abnormal HDL-C, HOMA-IR, hs-CRP and multiple cardiometabolic risk factors were more common among youth with severe obesity.

**Conclusions:**

Youth with severe obesity have identifiable differences in psychosocial and behavioral attributes that can be used to develop targeted intervention strategies to improve their health.

## Background

Obesity and severe obesity are prevalent in the United States and the rates continue to rise among children. Recent data from NHANES indicates that 18.5% [15.8–21.3] of youth ages 2–19 years have obesity, defined as body mass index (BMI) ≥ 95^th^ percentile of a sex-specific BMI-for-age, and 5.6% [4.0–7.6] have severe obesity, defined as BMI ≥ 120% of 95^th^ percentile of sex-specific BMI-for-age. Severe obesity increased from 4.0% in 1999–2000 to 6.0% in 2015–16 and the highest prevalence is seen among adolescents ages 12–19 years at 7.7% [5.0–11.2] [1, 2]. Severe obesity is disproportionately higher among minorities (Hispanics and Non-Hispanic Blacks), youth living in non-urban areas, and households headed by individuals with high school education or less [3].

Children and adolescents with severe obesity are at higher risk for abnormal cardiovascular risk factors including higher blood pressure, lipids, insulin resistance and diabetes [4–6]. Additionally, they are more likely to experience asthma [7], depression, anxiety, and poor psychological well-being [8]. There is a critical need to identify behaviorally-based interventions that will maximize health of youth with severe obesity [9–11]. Yet, few studies have identified psychosocial and behavioral factors for severe obesity [12] that could inform such strategies. This study aimed to examine the psychosocial, behavioral, and clinical correlates of severe obesity in a cohort of youth to identify the contextual and behavioral factors that differentiate those with severe obesity compared to those with non-severe obesity and overweight.

## Methods

The POOL registry cohort, named for the various participating clinics, was a prospective and voluntary registry for youth with overweight and obesity, ages 2–25 years old recruited from weight management and primary care clinics at Boston Children’s Hospital (BCH) from 2012 to 2016. The eligibility criteria were a) BMI ≥ 85^th^ percentile of a sex-specific BMI-for-age for those < 18 years on CDC 2000 growth charts or ≥ 25 kg/m^2^ for those ≥18 years; b) English speaking; and c) residents of New England as determined by the zip code of residence. Patients intending to undergo bariatric surgery for weight loss underwent the baseline visit prior to the surgery. Exclusion criteria were a) significant cognitive, psychosocial or medical illness that limits the subject’s ability to provide assent and/or participate in the research procedures such as severe autism, developmental delay and/or other conditions as determined by the investigators and b) pregnancy or intention to become pregnant within the next year for females. Patients were recruited by flyers posted in the clinics, by mail, or during a clinical visit. The study protocol was approved by the Institutional Review Board at BCH.

### Measures

Study visits were conducted in the Clinical and Translational Study Unit (CTSU) after an overnight fast. An average of two measurements of height (nearest 0.1 cm) and weight (nearest 0.1 kg) obtained using a fixed stadiometer and Tanita digital scale, respectively with no shoes worn and outer clothing removed were obtained. The BOD POD Gold Standard Body Composition Tracking System (COSMED, the Metabolic Company, Rome, Italy), an air displacement plethysmograph (ADP) method that measures whole body densitometry was used to determine fat and fat free mass [13, 14]. Three measurements of blood pressure were taken by auscultation in the seated position on the right arm after 3 min rest, and the average of the final two measurements was used. Physicians conducted Tanner staging by physical examination on participants ages 6 to 18 years [15]. Tanner stage 1 was considered prepubertal, and all others post-pubertal. Children < 6 years were assumed to be prepubertal and those > 18 years post-pubertal.

Parents of participants ≤12 years and older participants completed a) sociodemographic questionnaire with questions on race, ethnicity, and education, b) family history, c) medical history of conditions that were diagnosed by a health care provider including mental health diagnoses such as depression, anxiety, and ADHD, and d) questions adapted from the Youth Risk Behavior Questionnaire on diet, physical activity, sedentary behaviors, and attitudes about health [16]. A 10-point Likert scale question was used to assess the level of stress experienced by the youth in the past year where 1 means ‘no stress’ and 10 means ‘extreme amount of stress'. Pediatric Quality of Life Inventory (PedsQL) was used to measure health-related quality of life (HRQOL). The PedsQL has developmentally appropriate modules that assess 4 generic core scales (Physical, Emotional, Social, and Work or School functioning). Scores were calculated for each scale as well as for psychosocial health, which is an average of the emotional, social and work/school functioning scores. The overall health is the average of physical and psychosocial health scores. Parents completed the parent proxy age specific modules for participants ≤12 years; whereas, older participants completed the age-specific self-report modules [17, 18]. Participants ≥13 years completed items on the Three-Factor Eating Questionnaire (TFEQ-R18) to measure 3 aspects of eating behavior including cognitive restraint (CR), uncontrolled eating (UE), and emotional eating (EE) [19] and selected items from McKnight Risk Factor Survey to assess disordered eating, which included the following questions: “In the past 12 months, how often have you kept eating and eating and felt like you could not stop?” and “In the past 12 months, how often did you eat more than usual when you were bored or upset?” [20].

### Lab assay methods

Fasting blood samples were obtained for lipids, insulin, glucose and hs-CRP and tested within 24 hours. The tests assays were performed on Roche/Hitachi Cobas® c system analyzer using the following methods: 1) Triglycerides: lipoprotein lipase enzymatic test (CV range 0.7–2.0%); 2) High-density lipoprotein (HDL-C) and Total Cholesterol (TC): enzymatic colorimetric method (CV range: HDL-C: 0.5–1.5%; TC: 0.6–1.6%); 3) Low-density lipoprotein (LDL-C) cholesterol: Friedewald calculation (TC = HDL-C + LDL-C + VLDL-C where VLDL-C is defined as TG/5 in the fasting state); 4) Glucose: hexokinase enzymatic test (CV range 0.5–1.3%); 5) Insulin: electrochemiluminescence Immunoassay (ECLIA, CV range 1.1–4.9%); 6) hs-CRP: particle-enhanced immunological agglutination assay (CV range 0.6–3.5%).

### Analysis methods

All analyses are cross-sectional. BMI was calculated as weight/height^2^ (kg/m^2^). The definition of clinical and laboratory parameters used in the study is noted in Table 1. Descriptive statistics were calculated as the median and range for continuous variables and as proportions for categorical variables. Correlation between continuous measures was assessed using the Spearman coefficient. Proportions were compared among weight categories by Fisher’s exact test. Due to skewness, distributions of continuous variables were compared among the weight categories using the Jonckheere-Terpstra Test for Independent Samples. An analysis of co-variance (ANCOVA) was conducted to control for age and sex when comparing metabolic risk factor variables across weight categories. Log-transformation was performed for variables with significant departure from normality. A two -sided *p*-value < 0.05 was considered statistically significant. All analyses were conducted with IBM SPSS Statistics for Windows, Version 23.0 (IBM Corp, Armonk, NY).

**Table 1:** Definitions of cohort measurement parameters

	Description		Reference
**Weight categories**	**Age < 18 years**	**Age ≥ 18 years**	
Overweight	BMI 85-95^th^ percentile	BMI 25–30 kg/m^2^	CDC 2000 growth curves; Gulati 2012; Skinner 2015; Skinner 2016
Class 1 obesity	BMI 95–120% of 95^th^ percentile	BMI 30–35 kg/m^2^	
Class 2 obesity	BMI 120–140% of 95^th^ percentile	BMI 35–40 kg/m^2^	
Class 3 obesity	BMI > 140% of 95^th^ percentile	BMI > 40 kg/m^2^	
**Laboratory parameters**	**Definition of Abnormal**		
Fasting blood glucose	≥ 126 mg/dL	All ages	ADA diabetes care guidelines 2018
Fasting insulin	> 12 uIU/mL	All ages	Laboratory reference range
HOMA-IR	≥ 3 units	All ages	
C-reactive protein	≥ 3 mg/dL	All ages	Framingham Heart Study CHD Risk Scores; Jialal 2004; Buckley 2009
LDL	≥110 mg/dL	≥120 mg/dL	NHLBI Integrated Guidelines for Cardiovascular Risk Reduction in Children and Adolescents (Expert Panel, 2011)
Total Cholesterol	≥170 mg/dL	≥190 mg/dL	
HDL	≤ 40 mg/dL	All ages	
Triglycerides	≥ 75 mg/dL	ages 2–9 years	
	≥ 90 mg/dL	ages 10–19 years	
	≥ 115 mg/dL	ages ≥20 years	
SBP	≥ 95^th^ %tile for age, sex and height	ages 2–17 years	Fourth Report on the Diagnosis, Evaluation, and Treatment of High Blood Pressure in Children and Adolescents (2004) Seventh Report of the Joint National Committee on the Prevention, Detection, Evaluation, and Treatment of High Blood Pressure for adults (2003)
	≥ 140 mmHg	ages ≥18 years	
DBP	≥ 95^th^ %tile for age, sex and height	ages 2–17 years	
	≥ 90 mmHg	ages ≥18 years	
Multiple risk factor variable	Abnormal values for glucose, LDL, HDL, CRP or BP or taking medications for diabetes, dyslipidemia or hypertension		

## Results

A total of 803 potentially eligible children were identified from the 7 clinics and 681 were screened and eligible, of which 387 declined to participate; 102 could not be contacted again; 192 were eligible and agreed to participate; and 105 met all eligibility criteria and completed the baseline visit. Characteristics of the 105 children who participated in the baseline assessment are shown in Table 2. Median age of participants was 16.1 years (65% females); participants were of diverse racial and ethnic backgrounds. About half the sample (51.4%) had severe obesity, while 29.5% had non-severe obesity, and 19.1% had overweight. There were no statistically significant differences in the sociodemographic variables by category of weight. Median BMI of the participants was 31.7 kg/m^2^ (range 18.1–63.6) and median total body fat of 37.6% (range 12.2–61.0). Median total body fat increased significantly across the weight categories, and correlated strongly with BMI (r = 0.76; *p* = < 0.001). About half of participants were post-pubertal across each of the weight categories. There was a strong family history of obesity, with 75% of the participants reporting maternal obesity. Participants with severe obesity had a number of comorbid conditions, with asthma (26.7%) being the most common, followed by depression and obstructive sleep apnea, as compared to those with obesity or overweight. Amongst the less frequently self-reported conditions (data not shown), 1 participant had type 2 diabetes and 3 reported an eating disorder.

**Table 2:** Sociodemographic and clinical characteristics ofstudy participants overall and according to the weight category

Characteristic	*n*^a^	All	Weight Category			
			Overweight	Obese	Severely Obese	*p*-value**
Age; median (min, max)	105	16.07 (4.62, 25.54)	14.40 (4.62, 22.99)	14.40 (7.30, 21.98)	17.06 (5.17, 25.54)	0.18
Age Group; *n* (%)	105					0.42
Under 13 years		32 (30.5%)	6 (30.0%)	12 (38.7%)	14 (25.9%)	
13 years or older		73 (69.5%)	14 (70.0%)	19 (61.3%)	40 (74.1%	
Gender; *n* (%)	105					0.96
Male		41 (39.0%)	7 (35.0%)	12 (38.7%)	22 (40.7%)	
Female		64 (69.0%)	13 (65.0%)	19 (61.3%)	32 (59.3%)	
Race; *n* (%)	100					0.52
Caucasian		34 (34%)	6 (33.3%)	12 (41.4%)	16 (30.2%)	
African-American		44 (44%)	6 (33.3%)	13 (44.8%)	25 (47.2%)	
Multiple Races/Other		22 (22%)	6 (33.3%)	4 (13.8%)	12 (22.6%)	
Ethnicity; *n* (%)	102					0.58
Hispanic/Latino		22 (21.6%)	3 (15.8%)	5 (16.7%)	14 (26.4%)	
Not Hispanic/Latino		80 (78.4%)	16 (84.2%)	25 (83.3%)	39 (73.6%)	
Mother’s Education; *n* (%)	99					0.22
Some College or Less		60 (60.6%)	10 (55.6%)	15 (50.0%)	35 (68.6%)	
College Graduate or higher		39 (39.4%)	8 (44.4%)	15 (50.0%)	16 (31.4%)	
Father’s Education; *n* (%)	76					0.08
Some College or Less		54 (71.1%)	7 (46.7%)	16 (76.2%)	31 (77.5%)	
College Graduate or Higher		22 (28.9%)	8 (53.3%)	5 (23.8%)	9 (22.5%)	
BMI, kg/m2; median (min, max)	105	31.72 (18.05,63.61)	26.02 (18.05,29.26)	30.46 (20.15,34.48)	39.60 (24.63,63.61)	< 0.001
BMI z-score; median (min, max)	105	2.18 (1.14, 3.78)	1.39 (1.14,1.62)	1.91 (1.59,2.24)	2.49 (2.04,3.78)	< 0.001
% Total Body Fat	105	37.60 (12.2, 61.0)	31.75 (12.20,42.90)	35.60 (20.90,43.30)	45.60 (20.40,61.00)	< 0.001
Weight Categories; *n* (%)	105					
Overweight		20 (19.1%)				
Obese		31 (29.5%)				
Severely Obese		54 (51.4%)				
Tanner Stage; *n* (%)	99					0.20
Pre-pubertal		15 (15.2%)	2 (10.5%)	6 (20.0%)	7 (14.0%)	
Pubertal		29 (29.3%)	8 (42.1%)	11 (36.7%)	10 (20.0%)	
Post-pubertal		55 (55.6%)	9 (47.4%)	13 (43.3%)	33 (66.0%)	
Family History Obesity; *n* (%)						
Mother	84	63 (75.0%)	9 (56.3%)	16 (76.2%)	38 (80.9%)	0.19
Father	69	30 (43.5%)	5 (35.7%)	7 (41.2%)	18 (47.4%)	0.80
Medical Diagnoses; *n* (%)	105					
Asthma		28 (26.7%)	1 (5.0%)	6 (19.4%)	21 (38.9%)	0.007
High Cholesterol		19 (18.1%)	4 (20.0%)	8 (25.8%)	7 (13.0%)	0.33
Depression		17 (16.2%)	1 (5.0%)	2 (6.5%)	14 (25.9%)	0.02
Anxiety		13 (12.4%)	1 (5.0%)	5 (16.1%)	7 (13.0%)	0.58
ADHD		11 (10.5%)	1 (5.0%)	4 (12.9%)	6 (11.1%)	0.84
OSA		10 (9.5%)	0 (0%)	1 (3.2%)	9 (16.7%)	0.04
GE Reflux		10 (9.5%)	1 (5.0%)	1 (3.2%)	8 (14.8%)	0.20

^a^number of participants after excluding missing or don’t know/unknown responses for each variable

**P-values calculated from Fisher Exact Tests for categorical data or Jonckheere-Terpstra Test for Independent Samples for continuous data

Among the dietary and eating behaviors, self-reported frequency of intake of specific food and drinks did not differ significantly across the weight categories (Table 3). However, among the subset of participants ≥13 years, eating when bored was more frequently reported among those with obesity compared to those with overweight (*p* = 0.022). There was no significant difference in the eating behavior scores on the TEFQ-R18 for CR and UE by weight category; however, median scores for EE increased with increasing weight category (11.1 vs. 16.7 vs. 33.3 respectively, *p* = 0.026). Subjects with severe obesity were more likely to report playing no sports or organized activities compared to those without (47.2% vs. 35.5% vs. 15.8%, respectively, *p* = 0.044). Sedentary activities such as watching TV and video games or being active for ≥30 min/day were similar across the weight categories.

**Table 3:** Food intake and eating and activity behaviors according to weight category

Variable	*n*^a^	Weight Category			*p*-value**
		Overweight	Obese	Severely Obese	
***Food intake and eating behaviors among all participants***					
# servings per day **Water**, *n* (%)	102				0.31
1–3 /month to 1/day		5 (26.3%)	5 (16.7%)	6 (11.3%)	
2–3 /day		3 (15.8%)	11 (36.7%)	14 (26.4%)	
More than 3/day		11 (57.9%)	14 (46.7%)	33 (62.3%)	
# servings per day **Regular Soda**, *n* (%)	100				0.50
None		6 (31.6%)	10 (34.5%)	23 (44.2%)	
1–3 /month to 1/week		6 (31.6%)	11 (37.9%)	20 (38.5%)	
2–4 /week to more than 3/day		7 (36.8%)	8 (27.6%)	9 (17.3%)	
# servings per day **Diet Soda**, *n* (%)	102				0.96
None		13 (72.2%)	19 (63.3%)	36 (66.7%)	
1–3 /month to 1/week		4 (22.2%)	7 (23.3%)	12 (22.2%)	
2–4 /week to more than 3/day		1 (5.6%)	4 (13.3%)	6 (11.1%)	
# servings per day **Fruits**, *n* (%)	100				0.13
None		3 (16.7%)	0 (0%)	1 (2.0%)	
1–3 /month to 1/week		3 (16.7%)	5 (16.1%)	8 (15.7%)	
2–4 /week to more than 3/day		12 (66.7%)	26 (83.9%)	42 (82.4%)	
# servings per day **Vegetables**, *n* (%)	103				0.42
None		1 (5.6%)	1 (3.2%)	3 (5.6%)	
1–3 /month to 1/week		1 (5.6%)	8 (25.8%)	8 (14.8%)	
2–4 /week to more than 3/day		16 (88.9%)	22 (71.0%)	43 (79.6%)	
# days per week **eat dinner at home;** median (min, max)	102	4.0 (0,7.0)	4. 0 (0,7.0)	3.0 (0,7.0)	0.06
# days per week **eat fast food;** median (min, max)	103	1.0 (0,4.0)	2.0 (0,5.0)	1.0 (0,7.0)	0.19
# days per **week eat breakfast;** median (min, max)	95	6.0 (0,7.0)	7.0 (0,7.0)	5.5 (0,7.0)	0.72
***Eating behaviors among the subsample of adolescents 13 years and older***					
Eat when bored or upset; *n* (%)	69				0.022
Never to less than once a month		13 (100%)	11 (57.9%)	18 (48.6%)	
1 to 2 times a month		0 (0%)	4 (21.1%)	9 (24.3%)	
Once a week or more		0 (0%)	4 (21.1%)	10 (27.0%)	
Could not stop eating; *n* (%)	69				0.17
Never to less than once a month		13 (100%)	14 (73.7%)	26 (70.3%)	
1–2 times a month		0 (0%)	5 (26.3%)	9 (24.3%)	
Once a week or more		0 (0%)	0 (0%)	2 (5.4%)	
TFEQ-R18 Eating Behavior Scores (0–100); median (min, max)					
Cognitive Restraint (CR)	71	45.6 (0,59.1)	40.9 (0,68.2)	40.9 (13.6,77.3)	0.86
Uncontrolled Eating (UE)	71	29.2 (3.7,51.9)	40.7 (0,92.6)	40.7 (3.7,83.3)	0.11
Emotional Eating (EE)	70	11.1 (0, 66.7)	16.7 (0,100.0)	33.3 (0,100.0)	0.026
***Activity behaviors among all participants***					
# hours **watching TV** per day during weekdays; median (min, max)	103	3.0 (1.0,6.0)	3.0 (1.0, 7.0)	3.0 (1.0,7.0)	0.18
# hours **playing video games** per day during weekdays; median (min, max)	101	3.0 (1.0,7.0)	3.0 (1.0,7.0)	3.0 (1.0,7.0)	0.59
# days per week active **for 30 + mins/day**; median (min, max)	103	3.0 (1.0,7.0)	3.0 (0, 7.0)	3.0 (0, 7.0)	0.90
**# organized sports teams/physical activities** in the past year ; *n*, %	103				0.044
None		3 (15.8%)	11 (35.5%)	25 (47.2%)	
One		7 (36.8%)	9 (29.0%)	19 (35.8%)	
Two or more		9 (47.4%)	11 (35.5%)	9 (17.0%)	

^a^number of participants after excluding missing or don’t know/unknown responses for each variable

**P-values calculated from Fisher Exact Tests for categorical data or Jonckheere-Terpstra Test for Independent Samples for continuous data

The prevalence of abnormal levels of metabolic risk factors among all participants was high for lipids, hs-CRP, HOMA-IR, and insulin, whereas it was low for blood pressure and glucose (Table 4). Statistically significant differences in the distributions of these risk factors by weight category were observed for the HDL-C, HOMA-IR, Insulin and hs-CRP. In this study, BMI showed no correlation with total cholesterol (r = − 0.054; *p* = 0.588), LDL-C (r = 0.078; *p* = 0.432) or triglycerides (r = 0.161; *p* = 0.102) but a moderate inverse correlation with HDL (r = − 0.352; *p* <  0.001). The greatest difference was observed for hs-CRP; median hs-CRP for those with severe obesity was 3–4 times higher than those with overweight or non-severe obesity, and exceeded the cutoff for elevated risk of CVD (3.4 mg/L). Participants with severe obesity also had a significantly greater median number of risk factors that remained after adjusting for age and gender (data not shown)**.**

**Table 4:** Metabolic risk factors overall and according to weight category

Characteristic^a^	*n*^b^	% Exceed Cutoff	All	Weight Category			
				Overweight	Obese	Severely Obese	*p*-value**
Triglycerides, mg/dL	104	36.5%	79.5 (36.0, 362.0)	62.0 (36.0,362.0)	85.5 (46.0,241.0)	79.5 (43.0, 227.0)	0.26
Total Chol, mg/dL	104	43.3%	164.0 (118.0,237.0)	160.5 (122.0,237.0)	173.0 (124.00,228.0)	163.0 (118.0,231.0)	0.73
HDL-Chol, mg/dL	104	53.8%	45.0 (24.0, 76.0)	54.5 (26.0,76.0)	46.0 (24.0,69.0)	44.0 (24.0,76.0)	0.011
LDL- Chol, mg/dL	104	41.3%	96.0 (21.0,155.0)	88.0 (47.0,154.0)	100.0 (21.0,155.0)	96.5 (43.0,151.0)	0.40
SBP, mmHg	105	1.9%	110.0 (87.0, 140.0)	105.0 (90.0,125.0)	109.0 (95.0,140.0)	112.5 (87.0, 138.0)	0.051
DBP, mmHg	105	1.0%	67.0 (43.5, 85.0)	63.0 (57.0,81.0)	67.0 (43.5,85.0)	68.0 (47.5,81.0)	0.59
CRP, mg/L	104	28.8%	1.63 (0.10, 70.41)	0.97 (0.10, 3.85)	0.77 (0.18, 70.41)	3.40 (0.19, 38.16)	< 0.001
HOMA-IR	103	53.4%	3.08 (0.21, 23.61)	2.13 (0.21,5.65)	3.01 (0.52,8.05)	3.94 (0.69,23.61)	< 0.001
Insulin, uIU/mL	103	63.1%	16.0 (1.4, 102.8)	10.6 (1.4, 26.3)	11.6 (2.8, 35.2)	20.05 (3.8,102.8)	< 0.001
Glucose, mg/dL	104	2.9%	80.0 (62.0, 255.0)	80.5 (62.0,120.0)	82.0 (70.0,97.0)	79.0 (63.0,255.0)	0.54
**# of risk factors**	103		2 (0, 5)	1 (0,3)	1 (0,4)	2 (0,5)	0.003

^a^Median (Minimum, Maximum) values for each risk factor

^b^number of participants with non-missing data

**P-values calculated from Jonckheere-Terpstra Test for Independent Samples

Subjects with severe obesity were more likely to report having sub-optimal health habits and health status when compared to those without (27.5% vs.12.5% vs. 11.1%, respectively; *p* = 0.19 and 62.5% vs.37.9% vs. 35.3%, respectively; *p* = 0.053, Table 5). They also reported higher median levels of stress (6.5 vs. 6.0 vs. 5.0, respectively, *p* = 0.035). Although most quality of life measures were lower in both the severely obese and obese, there were no statistically significant differences or consistent trends by weight category (Table 5). A posthoc subgroup analysis of the data from youth older than 13 years (*n* = 68), who completed the survey themselves as compared to youth 12 years and younger (where parents completed the survey, *n* = 37) found that statistically significant differences in self-reported stress were seen in the older youth (*p* = 0.04), while in the younger children, lower scores were seen on social scale of PedsQL (*p* = 0.03) and approached significance in emotional scale (*p* = 0.05) and total score (*p* = 0.06).

**Table 5:** Health related attitudes and perceptions

Variable	*n*^a^	Overweight	Obese	Severely Obese	*p*-value**
General Health					
Health in General; *n* (%)	100				0.19
Fair/Poor		2 (11.1%)	4 (12.5%)	14 (27.5%)	
Good/Very good/Excellent		16 (88.9%)	27 (87.1%)	37 (72.5%)	
Health Habits; *n* (%)	94				0.05
Fair/Poor		6 (35.3%)	11 (37.9%)	30 (62.5%)	
Good/Very good/Excellent		11 (64.7%)	18 (62.1%)	18 (37.5%)	
Perceived Stress [1–10]	94	5.0 (1.0,9.0)	6.0 (2.0,10.0)	6.5 (2.0,10.0)	0.01
Quality of Life Scores (0–100); median (min, max)					
Overall	100	81.5 (48.9, 95.7)	75.0 (36.4,100.0)	75.0 (32.6,100.0)	0.11
Physical Health	102	87.5 (21.9,100.0)	78.1 (34.4,100.0)	78.1 (37.5,100.0)	0.15
Psychosocial Health	100	80.8 (53.3,95.0)	70.0 (37.5,100.0)	70.0 (26.7,100.0)	0.15
School Functioning	101	80.0 (45.0,95.0)	65.0 (30.0,100.0)	65.0 (25.0, 100.0)	0.26
Emotional Functioning	101	77.5 (45.0,100.0)	70.0 (25.0, 100.0)	65.0 (25.0, 100.0)	0.26
Social Functioning	100	85.0 (45.0,100.0)	90.0 (35.0,100.0)	80.0 (30.0,100.0)	0.16

^a^number of participants after excluding missing or don’t know/unknown responses for each variable

**P-values calculated from Fisher Exact Tests for categorical data or Jonckheere-Terpstra Test for Independent Samples for continuous data

## Discussion

In this cross-sectional, clinic-based cohort of youth with overweight and obesity, there were expected increases in % total body fat with increasing classes of obesity. While the individuals with severe obesity were slightly older, the differences were not statistically significant. Similarly, youth with severe obesity had a higher prevalence of co-morbidities including, asthma, OSA, depression, presence of multiple cardiometabolic risk factors, low HDL-C, elevated HOMA-IR and elevated hs-CRP. Psychosocial and behavioral phenotypic differences were most compelling and possibly interrelated, including higher rates of emotional eating, eating when bored and perceived stress with increasing severity of obesity, and lower participation in organized sports as well as less maternal education. Understanding the underlying biology and interrelationships between these psychosocial and behavioral factors may help us to develop more effective prevention and treatment interventions to improve the health of youth with severe obesity.

The adverse levels of HDL-C, hs-CRP and HOMA-IR in individuals with severe obesity are consistent with the findings from other studies. Lower levels of HDL-C have been identified in youth with severe obesity from population-based NHANES data at younger ages prior to identifiable differences in other lipid parameters [5]. Elevated levels of hs-CRP associated with obesity in adults [21], and thought to herald the onset of cardiometabolic consequences, such as coronary heart disease [22], hypertension [23, 24], metabolic syndrome [25, 26] and diabetes [27] were significantly higher in the youth with severe obesity compared to those without. While such levels have been previously identified in adults, a similar association has not been well defined in children [21]. The presence of elevated hs-CRP and HOMA-IR in youth with severe obesity adds to the growing body of evidence that supports an urgent call for intervention to prevent the potential oncoming wave of cardiometabolic disease in the future generations.

Similar to other studies, there was a higher proportion of asthma, depression and obstructive sleep apnea in individuals with severe obesity. In a systematic review, Papoustakis et al noted the association of asthma in 30 out of the 31 cross-sectional studies and 12 out of the 13 prospective studies of individuals with obesity [28]. While the exact mechanism of this association remains to be elucidated, proposed mechanisms include systemic inflammation and mechanical effect, both due to expansion of the adipose tissue. There may also be a contribution of insulin resistance and a role of intestinal microbial dysbiosis causing higher lipopolysaccharides or other inflammatory agents [29, 30]. The association of depression with severe obesity has also been described, albeit without a clear directionality. One representative study of adolescents with obesity found that after adjusting for demographics and emotional eating, the odds of having severe obesity versus obesity were 3.5 times higher for patients with depression (as measured by PHQ-9; a score ≥ 11 was considered depression) compared with those without (OR = 3.5, 95% CI 1.2,20.9, *p* = 0.030) [31]. Similarly, the odds of having severe obesity were also higher with anxiety (Generalized Anxiety Disorder Scale ≥10, OR = 4.93, 95% CI = 1.17,20.85, *p* = 0.030). In this study, there was no association of either depression or anxiety with emotional eating, but other studies have found emotional eating as a mediator for obesity in individuals with depression [32, 33]. A high level of psychosocial dysregulation along with problematic eating patterns and behaviors was noted by Gowen et al in their study of 54 young adults and adolescents [34]. Similarly, in a comparable group of adolescents prior to bariatric surgery, higher BMI, depressive symptoms and the number of medical comorbidities were significant predictors of the impaired weight related quality of life and closely related to dysregulated eating behaviors [35]. Differences in our findings that relate depression and anxiety to eating behaviors may be due to the use of different assessment strategies, as our study used medical diagnoses of depression or anxiety reported by the parent or adolescent/young adult rather than questionnaire measures that assess current depressive or anxiety symptoms. It is also postulated that the presence of depression elicits a response from the hypothalamic adrenal axis that leads to inflammatory milieu leading to obesity [36].

Notably, this study found that emotional eating and eating when bored were increased in individuals with severe obesity. A body of literature demonstrates the association of emotional dysregulation with weight gain and obesity [12, 31, 37, 38]. It has been suggested in adults that a poor emotional regulation may entail the use of maladaptive strategies to manage emotions and stress, for example, by using highly palatable and energy dense food to suppress emotions [39]. Studies in children have demonstrated that emotional eating is often followed by negative emotions [40–43], and is likely to be a learned behavior [44, 45]. College students identified boredom as the emotion that most commonly triggers eating [46], a finding that was replicated in the laboratory where the completion of a boring task was associated with snacking desire [47]. Assessment of subtypes of emotional eating in a cohort of 189 adults showed eating in response to depression (EE-D), anxiety/anger (EE-A) and boredom (EE-B) related to poorer psychological well-being, greater symptoms of eating disorder and more difficulties with emotional regulation [48]. It is also possible that the association of depression and severe obesity is mediated by emotional eating as demonstrated in college students in Mexico City [32] and Netherlands [33]. Findings highlight the importance of building skills for emotional regulation for children and adolescents with severe obesity [49]. Findings for emotional eating could also be explained by experiences of weight stigma (e.g. weight related teasing) that are more likely to be experienced by youth with severe obesity [50].

This study highlights the role of stress experienced by youth with obesity, especially in the older age group, that is increasingly being recognized to have a complex, perhaps bidirectional, relationship [51]. Studies of hair cortisol concentration, thought to be deposited over longer periods of time, both in adults [52, 53] and in children [54] have been shown to have high associations with obesity. Whether this relationship is linked to biological factors such as higher levels of circulating glucocorticoids or higher response to circulating glucocorticoids that predispose individuals to weight gain, or changes in internal milieu, such as imbalances in glucose homeostasis, is not clear. On the other hand, the higher levels of weight stigma carried by individuals with obesity, mental health issues (e.g. depression, anxiety etc), or physical disorders (e.g. asthma, OSA) can lead to chronic stress and/or higher circulating corticosteroids, making it a vicious cycle. The presence of higher levels of co-morbidities that can lead to increased levels of biological stress as well as the higher perceived levels of stress in this study, emphasize the role of both recognition and management of stress in youth with obesity. The differences in the PEDS-QL in children with severe obesity, as have been noted in prior studies of children with obesity [55] were more prominent in the younger age group in this study, perhaps indicating that older children and adolescents may perceive the psychosocial pressures induced by obesity as stress.

Participants with severe obesity exhibited a reduced number of organized physical activities. It is not known whether the inactivity preceded the severe obesity or is a consequence of potential reduction in the agility of the body by excessive weight. However, there is evidence in the literature that lack of physical activity may be linked to the state of emotional dysregulation known to be associated with obesity [8, 48]. Emerging evidence emphasizing the positive role of team sport participation on longer term mental health, especially in children who have experienced adverse childhood events [56] makes this an important consideration in youth such as those in this study. This study found no statistically significant differences in the dietary intake of foods shown to be related to risk of obesity including the servings of water, sugar sweetened beverages, fruits and vegetables, as well as the patterns of meals eaten at home and fast food intake. The lack of differences may be due to the source of the sample, as participants were attending weight management programs and following treatment recommendations provided those programs.

This study is limited by its cross-sectional design as well as recruitment in tertiary care weight management programs that may limit the generalizability of some of the findings. While a thorough assessment of body composition and cardiometabolic risk factors was done, the measurement of dietary intake was limited to self-reported frequency of selected food groups and physical activities. Parent report on surveys for younger children limits our ability to discern the extent to which this represents children’s perceptions on various measures such as quality of life and stress. Further, the small sample size limits the comparison across gender and race/ethnicity groups. However, the comprehensive assessment in the study has provided the opportunity to identify important psychosocial, behavioral and clinical correlates that may play a role in the more effective management of obesity in such a population.

This study is one of the few studies that have tried to comprehensively characterize differences in physiological, psychosocial and behavioral phenotypes in adolescents with severe obesity. The higher prevalence of psychological and behavioral phenotypes in this sample suggests that there are modifiable and possibly related targets of intervention. It also re-emphasizes the need for a multi-disciplinary team including clinician, behavioral psychologist, social worker and exercise physiologist to identify and address multitude of complex problems in these patients. Future larger and longitudinal studies are needed to tease out the causal pathways and interactions between these important modifiable targets of intervention.

## Conclusions

Higher rates of co-morbidity were seen in youth with severe obesity compared to those without. Youth with severe obesity also had higher proportion of psycho-social and behavioral phenotypes including higher rates of emotional eating, eating when bored and perceived stress. Whether these are the causes or the results of severe obesity in youth needs to be explored further for better care of this growing population.

## Data Availability

The datasets generated and/or analyzed during the current study are not publicly available due to IRB restrictions for sharing individual level data but are available from the corresponding author on reasonable request.

## Abbreviations

POOL registry: Registry with patients enrolled from the PREP clinic, Optimal Weight for Life program, One Step Ahead program and Lipid clinic, Boston Children’s Hospital
BMI: Body Mass Index
HOMA-IR: Homeostatic Model Assessment of Insulin Resistance
hs-CRP: High sensitivity C-reactive protein
NHANES: National Health and Nutrition Examination Survey
CDC: Centers for Disease Control
ADHD: Attention Deficit Hyperactivity Disorder
HDL-C: High-density Lipoprotein Cholesterol
LDL-C: Low-density Lipoprotein Cholesterol
VLDL-C: Very low-density Lipoprotein Cholesterol
TG: Triglyceride
CV: Coefficient of variation
CVD: Cardiovascular disease
PHQ-9: Patient Health Questionnaire − 9
OSA: Obstructive Sleep Apnea
